# What causes medication administration errors in a mental health hospital? A qualitative study with nursing staff

**DOI:** 10.1371/journal.pone.0206233

**Published:** 2018-10-26

**Authors:** Richard N. Keers, Madalena Plácido, Karen Bennett, Kristen Clayton, Petra Brown, Darren M. Ashcroft

**Affiliations:** 1 Centre for Pharmacoepidemiology and Drug Safety, Division of Pharmacy and Optometry, School of Health Sciences, Manchester Academic Health Sciences Centre (MAHSC), University of Manchester, Manchester, United Kingdom; 2 NIHR Greater Manchester Patient Safety Translational Research Centre, MAHSC, University of Manchester, Manchester, United Kingdom; 3 Medicines Management Team, Greater Manchester Mental Health NHS Foundation Trust, Park House Hospital, North Manchester General Hospital, Manchester, United Kingdom; 4 School of Health and Human Sciences, University of Bolton, Bolton, United Kingdom; Nord University, NORWAY

## Abstract

**Objective:**

Medication administration errors (MAEs) are a common risk to patient safety in mental health hospitals, but an absence of in-depth studies to understand the underlying causes of these errors limits the development of effective remedial interventions. This study aimed to investigate the causes of MAEs affecting inpatients in a mental health National Health Service (NHS) hospital in the North West of England.

**Methods:**

Registered and student mental health nurses working in inpatient psychiatric units were identified using a combination of direct advertisement and incident reports and invited to participate in semi-structured interviews utilising the critical incident technique. Interviews were designed to capture the participants’ experiences of inpatient MAEs. All interviews were transcribed verbatim and subject to framework analysis to illuminate the underlying active failures, error/violation-provoking conditions and latent failures according to Reason’s model of accident causation.

**Results:**

A total of 20 participants described 26 MAEs (including 5 near misses) during the interviews. The majority of MAEs were skill-based slips and lapses (n = 16) or mistakes (n = 5), and were caused by a variety of interconnecting error/violation-provoking conditions relating to the patient, medicines used, medicines administration task, health care team, individual nurse and working environment. Some of these local conditions had origins in wider organisational latent failures. Recurrent and influential themes included inadequate staffing levels, unbalanced staff skill mix, interruptions/distractions, concerns with how the medicines administration task was approached and problems with communication.

**Conclusions:**

To our knowledge this is the first published in-depth qualitative study to investigate the underlying causes of specific MAEs in a mental health hospital. Our findings revealed that MAEs may arise due to multiple interacting error and violation provoking conditions and latent ‘system’ failures, which emphasises the complexity of this everyday task facing practitioners in clinical practice. Future research should focus on developing and testing interventions which address key local and wider organisational ‘systems’ failures to reduce error.

## Introduction

Mental health illness is common and has a significant detrimental impact on societies and national economies [1, 2]. Patients with these illnesses are a complex and vulnerable patient group with a high risk of premature mortality [3]. They are known to have high burdens of physical co-morbidity [4], do not always access appropriate health care and may suffer inequalities in the care they are provided [5, 6].

The use of medicines to treat mental illness is a mainstay of modern practice [7]. However, psychotropic polypharmacy [8–10], unlicensed psychotropic prescribing [11], and use of high risk medications such as lithium, depot antipsychotics and clozapine [12] are common in this population and present unique challenges for safe medication use. To compound the risks to patient safety that these issues create, prescribed medications may frequently change during psychiatric hospital admission [13] and not all mental health service users may feel involved in decisions about their drug treatment [14]. The way medicines are managed in hospital settings may also differ to general hospitals; for example in the United Kingdom (UK) patients may receive medication from a trolley located centrally and trained ‘runners’ such as health care assistants may take medication dispensed by nurses to patients on the ward [15, 16]. There is also emerging evidence that medicine-related factors may play a role in causing re-admission to mental health hospitals [17, 18]. This clearly highlights the importance of studying the quality and safety of medicines use in mental health settings, and there is growing international consensus on this issue [19, 20].

A recent systematic review of medication safety in mental health hospitals found that prescribing errors and medication administration errors (MAEs) were common in this setting, and that patients were often harmed by preventable medication related adverse events [21]. Indeed, MAEs may be the most commonly reported medication error in mental health hospitals [22, 23], with direct observation studies finding MAE rates of 3.3–48% of doses administered or omitted [24–26].

Whilst there is emerging evidence of the prevalence and nature of MAEs in mental health hospitals, an in-depth understanding of the underlying causes of these errors remains absent [21]. This is an important knowledge gap in the current evidence base, as the causes of MAEs in mental health hospitals may be different to general hospitals and a clearer understanding of the underlying causes of MAEs in this specialist setting could inform the design of effective interventions [27]. To date, available evidence of MAE causation in mental health settings is limited to quantitative analysis of observation data [24–26] (which cannot capture internal mental processes like intention), incident report reviews [22, 23, 28] (which often report limited descriptions of MAE causation) and more general explorations of medicines administration practices [15, 16] or related knowledge/competency [29]. Whilst these studies are useful in identifying some of the important MAE antecedents, including those that might differ to general hospitals (e.g. ‘runners’ [22, 23] and disturbed patients [22]), they do not provide a comprehensive account of the causal chain leading to error, nor how various factors may interconnect under different circumstances to lead to MAEs [27, 30]. Therefore, this study aimed to investigate in-depth the underlying causes of MAEs in a mental health National Health Service (NHS) hospital.

## Methods

### Definitions

Medication incidents were included in the study if they met the following definitions. Medication administration errors (MAEs) were defined as ‘a deviation from the prescriber’s order as written on the patient’s chart, manufacturer’s instructions or relevant institutional policies’ [27, 30]. Our definition of an MAE encompassed ‘near misses’ that were prevented from reaching the patient by any means [31]. Our analysis of the underlying causes of MAEs was informed by established human error theory, the components of which are defined later in this manuscript (see ‘Data Analysis’).

### Setting

Student and registered mental health nurses (RMNs) were recruited between March-July 2015 from one mental health hospital in the North West of England. The hospital had a total of 9 adult inpatient units catering for a variety of mental health illnesses including acute care, intensive care, later life and rehabilitation.

At the time the study was carried out, the study hospital utilised paper-based prescribing and an electronic patient record system. Medicines administration involved patients attending the ward clinic room to receive their medications (except later life wards). Medications were prepared for individual patients sequentially during medication rounds; pre-preparing medications or preparing multiple medications simultaneously for different patients was not recommended. Most oral medicines were authorised to be administered by a single qualified nurse, but in practice a second ‘runner’ (often a trained health care assistant or nurse) was frequently utilised to bring patients to the clinic room or to take medications to the patient directly. A group of ‘high risk’ medications including all injectable medicines (e.g. insulin, depot antipsychotics) and controlled drugs required two authorised members of staff to check and sign for administration; this most often involved two qualified nurses. Student nurses were only permitted to administer medications to patients under the direct supervision of a registered (qualified) nurse.

### Recruitment

Both student nurses and RMNs were eligible to participate provided they were working on mental health inpatient units at the time the MAE was made and were willing to discuss the causes of at least one of these incidents that they had been directly involved with. Near misses were included as MAEs as they also provide windows into the safety of systems, and student nurses were considered eligible to take part alongside RMNs in order to provide further diverse perspectives on what factors influence medication administration errors.

Participants were recruited to the study in three ways:

- Nursing staff who recorded MAEs using the local incident reporting system during and up to 6 months before the study began were identified and approached by the research team. This system allowed those reporting incidents to be identified by senior management and medicines safety staff for improvement, quality control and governance purposes,
- Local members of the medicines management team who identified MAEs on their inpatient wards reported these to the research team, who then approached the nursing staff member involved, and
- Members of the research team directly advertised the study to inpatient nursing staff during ward visits and using posters located in clinical ward areas.

Potential participants were then offered information packs (cover letter and participant information sheet) about the study. Anyone then interested in taking part contacted a member of the research team to arrange an interview. Written consent was provided before interviews took place.

### Data collection

The interview guide used in this study was adapted for the mental health setting following successful use by the research team investigating the causes of MAEs in UK general hospitals [30]. The interview guide can be found in the Appendix (S1 Appendix). Briefly, participants were asked to provide basic background demographic information before discussing one or more MAEs that either reached or did not reach the patient (near misses) that they were directly involved with. Emphasis was placed on recalling in detail the circumstances of the incident and what the participant thought caused it to happen. The interviews closed with participants providing insights into what they felt could have prevented their error from occurring.

Research team members RNK and MP conducted interviews face-to-face and following a semi-structured format with each participant. Each interview was digitally audio-recorded (with the exception of one interview, where the participant preferred written notes to be made instead) and lasted between 10–42 minutes. Research team members RNK and MP were both trained in qualitative interviewing and co-interviewed 3 participants (with their permission) in order to achieve consistency in interview style.

The design of the interview guide and overall approach to the interviews was based on the critical incident technique (CIT) [32]. The CIT has been successfully applied to help understand the causes of inpatient prescribing [33] and medication administration errors [30, 34], and facilitates the collection of rich and relevant data from otherwise busy ward staff by focusing on exploring their specific intentions, behaviours and actions when a particular error or near miss occurred [35].

### Data analysis

Interviews were transcribed verbatim. Transcripts were organised and coded using the NVivo computer software program (v10) according to the Framework method [36]. Framework is a seven step analytical approach designed to be systematic and transparent so that the process of translating raw data into emerging themes is clear [37]. Data are organised in matrices and summarised in individual cells so they can be reduced and compared across participants without losing context within individual accounts. The stages of analysis are as follows: the interviews are transcribed and researchers become familiar with it; then the data are coded and the conceptual framework is developed before being tested and applied; and finally the data are summarised in matrices and interpreted (e.g. by connecting themes/cases, or by developing typologies). Researchers are free to move between these analytical stages as required to refine concepts [36, 37].

**Fig 1:**
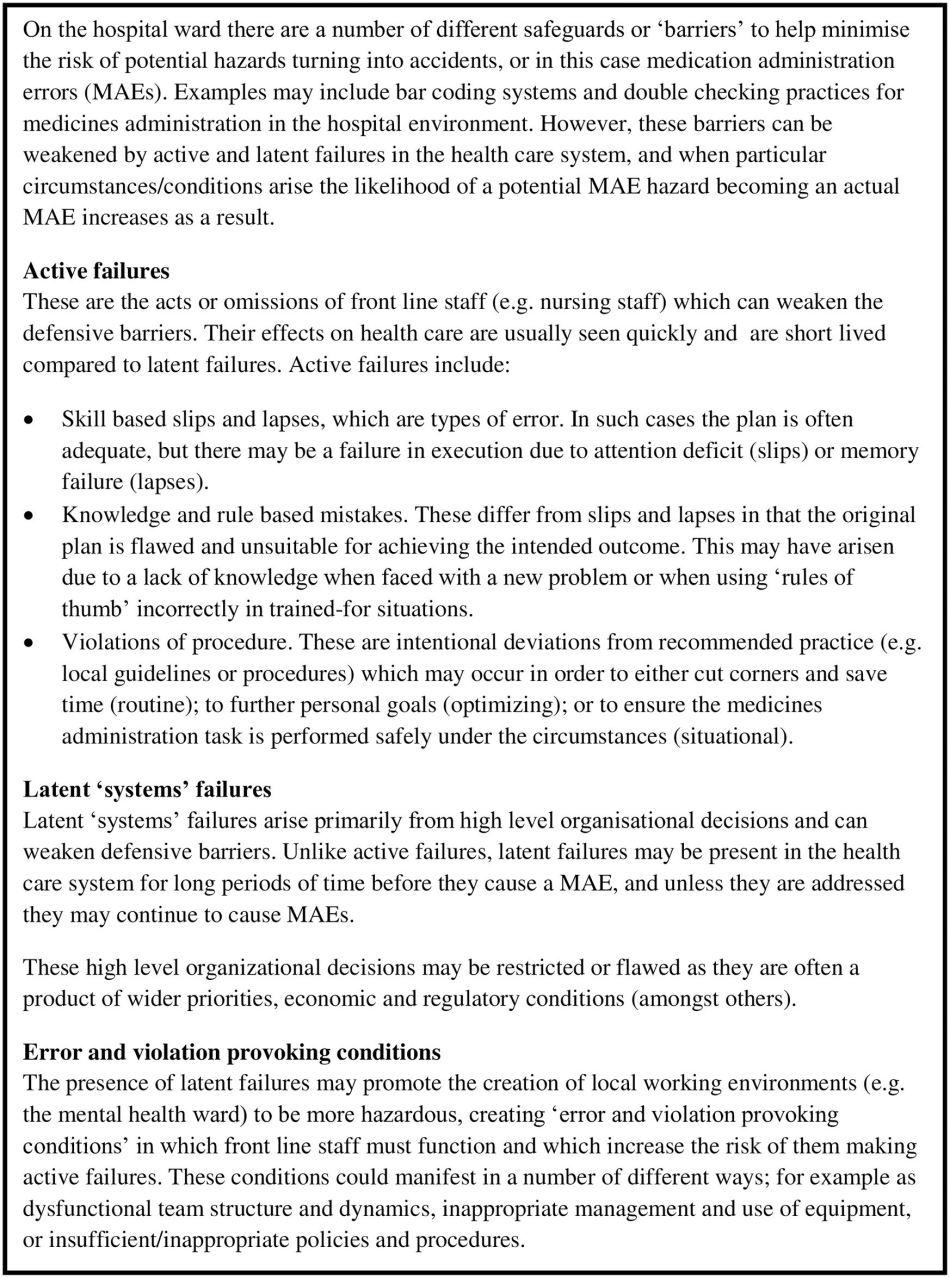
Reason’s model of accident causation applied to MAE research [27, 30, 38, 39].

The analysis framework was informed by Reason’s model of accident causation [38, 39]. Earlier research has applied this model to understand the causes of medication errors [27, 30, 33, 40], and it has been described in the context of MAE research in detail elsewhere (including summary diagrams of how Reason’s model applies to MAE research) [27, 30]. Reason’s model is summarised in Fig 1. Data were coded as active failures, error/violation provoking conditions and latent (wider organisational) failures, and one author (KB) independently extracted and analysed data for 10 interviews in order to confirm the suitability of the coding framework. Each MAE type (e.g. dose omission) was assigned by the research team [30]. Active failure assignments were confirmed following review and discussion between RNK and DMA.

### Ethical approval

The study was approved by the University of Manchester Research Ethics Committee 2 (submission 15104) and by the research and development department at the participating NHS trust.

## Results

**Fig 2:**
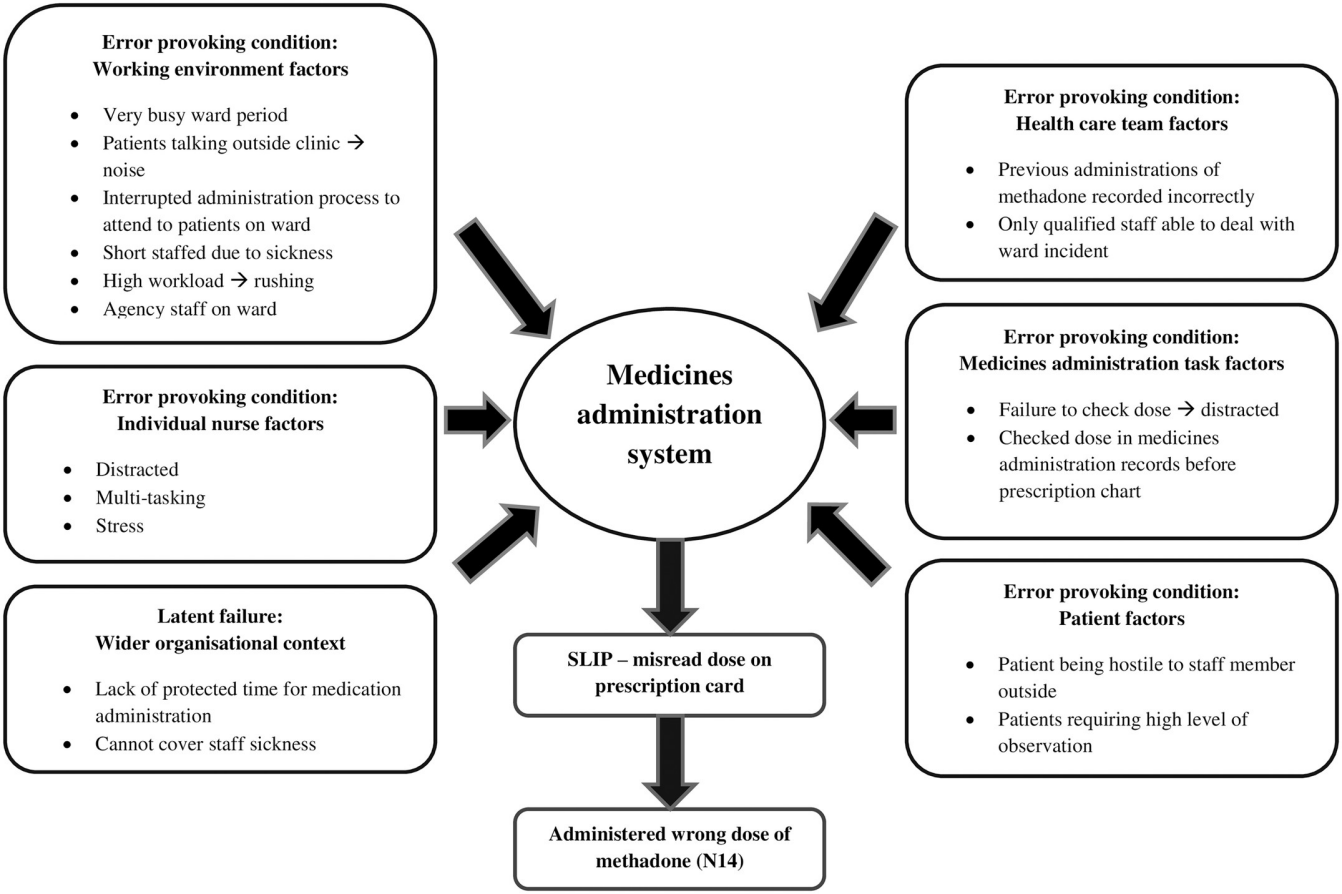
Summary of causes for one skill-based ‘slip’ medication administration error.

Twenty participants described a total of 26 MAEs, including 5 ‘near misses’ that did not reach the patient. No errors resulted in patient harm. A summary of interview participants and the MAEs they described can be found in Table 1. The majority of errors involved medications used to treat central nervous system (CNS) disorders (n = 21), and most commonly involved either wrong drug (n = 7), wrong patient (n = 5) or wrong dose errors (n = 4). The reported MAEs involved nurses of varying levels of experience post-qualification, with over half occurring when participants had 1 year or less (n = 14, 54%) experience (including 2 student nurse errors) and 7 (27%) when nurses had more than 5 years’ experience. Each error and near miss (active failure) was preceded by combinations of underlying error and violation provoking conditions, as exemplified in Fig 2 that describes the active failures and error provoking conditions underpinning one MAE discussed in this study.

**Table 1:** Participant information and incident summary.

Interview	Gender	Location of incident (inpatient unit)	Time since qualification when incident occurred	Error or near miss	Nature of incident	Drug class involved in incident	Route	Active failure
N01	F	Acute adult	< 1 year	Near miss	Gave unordered drug (discontinued)	Benzodiazepine	Oral	Slip
N02	F	Rehabilitation	> 5 years	Error	Wrong documentation	Calcium channel blocker	Oral	Lapse
		Acute adult	< 1 year	Error	Wrong drug	Depot antipsychotic	IM	Lapse
N03	F	Acute adult	< 1 year	Error	Wrong dose	Depot antipsychotic	IM	KBM
		Rehabilitation	> 5 years	Near miss	Wrong drug	Anti-manic	Oral	Slip
N04	M	Acute adult	NS, but > 1 year	Error	Wrong drug	Antipsychotic	Oral	Slip
N05	M	Rehabilitation	1–5 years	Near miss	Wrong dose	Antipsychotic	Oral	Slip
N06	F	Later Life	1–5 years	Error	Extra dose	Antipsychotic	Oral	Slip
		Later Life	NS	Error	Wrong patient	Laxative	Oral	NK
N07	F	Acute adult	< 1 year	Error	Wrong patient	Benzodiazepine	Oral	NK
N08	M	Acute adult	1–5 years	Error	Omitted dose	Opioid analgesic	Oral	KBM
N09	F	NS	Student nurse	Near miss	Wrong dose	Antidepressant	Oral	RBM
N10	F	Forensic	< 1 year	Error	Wrong time	Antihistamine	Oral	KBM
N11	M	Intensive care	> 5 years	Error	Wrong patient	Benzodiazepine	Oral	Lapse
N12	F	Acute adult	> 5 years	Error	Wrong patient	Multiple medications (psychotropic and non-psychotropic)	Oral	NK
N13	F	Treatment suite	> 5 years	Error	Wrong drug	Depot antipsychotic	IM	Slip
N14	F	Acute adult	> 5 years	Error	Wrong dose	Opioid dependence drug	Oral	Slip
		Acute adult	> 5 years	Error	Wrong drug	Opioid analgesic	Oral	Slip
N15	F	Acute adult	< 1 year	Error	Wrong formulation	Antipsychotic	Oral	Slip
N17	F	NS	Student nurse	Error	Wrong drug	Anti-manic	Oral	Slip
N18	F	Acute adult	< 1 year	Error	Gave unordered drug (discontinued)	Benzodiazepine	Oral	Slip
N19	F	Rehabilitation	1–5 years	Error	Omitted dose	Calcium channel blocker	Oral	Slip
N20	M	Acute adult	1–5 years	Error	Wrong patient	Thyroid replacement	Oral	Violation
		Intensive care	<1 year	Error	Wrong drug	Anti-manic	Oral	Slip
N21	F	Later life	<1 year	Error	Extra dose	Dopamine precursor	Oral	KBM
		Later life	1–5 years	Near miss	Wrong preparation	Benzodiazepine	IM	NK

F = female; IM = intramuscular; KBM = knowledge based mistake; M = male; NK = not known, other person prominently involved; NS = not specified; RBM = rule based mistake

### Active failures

Sixteen of the MAEs reported by participants involved skill based slips and lapses (n = 13 slips), with the remainder either knowledge (n = 4) or rule based mistakes (n = 1), and violations (n = 1). Some events could not be categorised as active failures as another staff member was more prominently involved (n = 4).

#### Slips and lapses

Skill based errors often resulted in nurses selecting the wrong product (drug or dosage) or overlooking or misinterpreting important information. One experienced nurse described how she administered co-codamol instead of codeine whilst busy and could only recall that the patient was prescribed co-codamol previously:

“… but the gentleman in question had been on co-codamol since he’d been here, and he’d only just been changed to codeine. So, in my head, I saw the codeine, but because I was that busy I thought, oh he’s been on co-codamol since he’s got here, so that’s why I gave him it [co-codamol].”

(N14, > 5 years’ experience)

#### Knowledge and rule based mistakes

In contrast to skill based errors, knowledge and rule based mistakes involved flawed planning. In one account, a junior nurse was not certain of her knowledge when faced with a novel problem:

“I think because I was newly qualified and it [how to dose ‘when required’ medications over 24 hours] hadn’t really been explained very well, so I thought that they could have two [doses] in a day so, say, today they could have two today, and then two lots tomorrow. But, you have to do it from the time, don’t you…”

(N10, < 1 year)

#### Violations

There was one reported violation, which involved the administration of levothyroxine to the wrong patient after a nurse who did not routinely work on the ward in question misidentified a service user using only their name and not their date of birth as recommended in the local policy for medicines administration.

### Error and violation provoking conditions

#### The health care team

Problems with written and verbal communication were discussed by 11 participants, and commonly led to skill-based errors and mistakes when staff missed, misinterpreted or made assumptions about information whilst working under challenging conditions (such as high workload, noisy environments, and distractions from staff or patients).

Written communication problems concerned prescriptions that were ambiguous because they had parts crossed out or were unclearly written, as well as previous medicines administration records that were not correct or were omitted. Problems with verbal communication were less common (n = 4 errors) but commonly involved communication breakdown between two staff members (e.g. between nurse and ‘runner’ who administered medication to the patient) and the use of using only the patient name for identification.

Three nurses discussed how a close working relationship with colleagues led to errors when working together to administer medications, with one experienced nurse revealing how he made an assumption about which patient was being discussed with his trusted colleague before making a skill based error and administering medication to the wrong patient:

“… I presumed that he [nursing colleague] knew I was talking about somebody else [medication to be administered], […] but I was just working on the assumption that he knew what I was thinking, which is quite strange really. Looking back, with hindsight, it seems stupid, but I suppose when you’re busy and you’re rushing, then it’s just one of those things.”

(N11, > 5 years)

Supervision and support were identified as an important barriers to error by participants, as deficiencies in these areas were frequently raised by participants as contributing to their errors or near misses, and by junior staff members in particular (though two examples of different errors occurred despite direct support). Participants discussed making both skill-based and planning errors as a consequence. Lone working was frequently implicated in these errors as this often caused anxiety which distracted participants, added to their workload if there were certain tasks only qualified nurses could perform (e.g. obtaining money from the safe) and gave them limited options for clarification in cases of uncertainty. One recently qualified nurse described how lone working during medicines administration limited her options for clarification which contributed to a knowledge based mistake:

“And, as a newly qualified I was left on my own, like, the only qualified nurse… […] … I wasn’t able to ask another nurse, I wasn’t able to check with them, because I was just on my own. Obviously, the patient was getting really upset and agitated, so I think that was probably another reason why I gave it [dose] a bit early.”

(N10, < 1 year)

#### The individual nurse

The majority of participants highlighted how considerable stress, nervousness (particularly amongst junior nurses) and feelings of pressure to complete tasks contributed to their errors and near misses; these mental states were driven primarily by high workload, low staffing, inexperience, patient acuity and inadequate skill mix and led to inappropriate decision making and skill based errors through lack of concentration or rushing.

Along with undergraduate students, new or recently qualified nursing staff in particular described how their unfamiliarity with certain medications (e.g. depot antipsychotics) and patients contributed to errors. Two nurses commented on how semi-sodium valproate was used “a lot more” (N03, > 5 years) on their wards compared to sodium valproate, which contributed to misidentification of these medicines through prior expectations.

When left on their own, junior nurses reported burdensome feelings of responsibility when working in isolation and questioned their readiness to cope with this role which put further pressure on themselves, causing mental distraction and flawed decision-making.

“I think I was naturally more anxious for that shift anyway because I was the only nurse on [the ward] and it was the first time so you’ve got that…I don’t know if it’s relevant but what I got was getting really anxious, like, what if I can’t manage it, I’ve only been qualified a few months, what if something goes wrong and it comes back on me, I’m looking after 20 patients on my own, I haven’t been qualified long. So I think there was an underlying anxiety as well that maybe I was also distracted by that when I was dispensing the medication which did probably have an effect as well.”

(N01, < 1 year)

Greater experience was highlighted as a protective barrier to error by one nurse who commented on how this gave them confidence to challenge patient demands in the interests of safety, rather than trying to respond immediately (and rushing) to meet their requests:

“… I’ve learnt over the years that some things don’t have to be done immediately, you know, you have to prioritise and especially where medicines are concerned, you need to do it right, so if it means taking an extra half hour, then unfortunately I’d just explain to the patient, you’re just going to have to wait half an hour before you can go on your leave.”

(N02, < 1 year)

#### The patient

Patient mental illness, behaviours (e.g. agitation, hostility) and requests contributed to every active failure. One nurse described how a lack of ward staffing meant that patients were not receiving attention and were instead interrupting staff with requests, leading to a skill-based slip:

“… there wasn’t enough staff and the patients obviously weren’t getting probably the attention that they wanted, so they did keep coming [to see staff], because some patients are demanding and they do want a lot of time off staff, which you can’t always give if you’re busy.”

(N06, 1–5 years)

Another participant who reported a violation described how a patient’s mental illness meant they did not intervene despite being addressed with the wrong name and given incorrect medications:

“But I remember I think because I was talking to the patient and saying their name, and over and over, and saying how are you doing today, da-da-da, and talking to them, I think the patient was very withdrawn and didn’t really talk too much with me and never really corrected me, I just assumed that that was fine and that that was the [correct] person.”

(N20, 1–5 years)

Other participants revealed how persistent demands from often agitated and aggressive patients led to them yielding to these requests despite their own uncertainties, due in part to lack of staff support/lone working and inadequate confidence, experience and/or knowledge. This was exemplified by one account of a wrong drug error involving a depot antipsychotic when the participant was a junior nurse, where patient distraction combined with multi-tasking, workload pressures and a change in prescription contributed to the pathway to error:

“I think I wasn’t familiar with the [depot] chart, because they changed, I let the patient mither [pester] me, she was, like, I want to go [leave the ward], I want to go, can you do it? And I was trying to do two things at once and because it was a depot [antipsychotic administration], it’s outside of your normal medicine round, so I think I was under other pressures and I was, like, oh, just get her depot done and then she can go on her leave, sort of thing, then it’s one less person around my feet asking for something, which is wrong, I can see that now, but at the time, you know, it’s quite easy to get drawn into…and really what I should have done to her was said, you’re just going to have to wait five minutes, just have a seat, let me just go and…and then I think if I’d have just took that extra couple of minutes, I wouldn’t have picked the wrong [medication] card up.”

(N02, < 1 year)

#### The working environment

The majority of participants (n = 17) described working environments that were noisy, chaotic, and/or busy. Some participants used the term “acute” (N15, < 1 year) to describe the ward conditions, which highlighted instances where there were multiple unwell/agitated patients or general disruption present. Many described how patients queued outside the clinic room to receive their medication, creating noise and asking questions of staff. This type of environment led to distractions and interruptions, high workload and rushing, and when combined with other factors led to both skill-based and planning errors as nurses were not able to focus on the medicines administration task.

“Rushed, there were lots of people milling around in the corridor, there were doctors running on and off the ward requesting prescription charts, there were medical students who were doing bloods on another patient in the clinic at the same time, so it was really crowded, it just felt really…just really rushed, I had to get the medication round finished to get on with the rest of the day […] One of the medical students was asking me where the blood bottles and needles were.”

(N01, < 1 year)

Distractions and interruptions were implicated in more than half of the incidents reported and the majority resulted in skill-based errors. Their origins were multifactorial, including high workload, patient factors, staff/skills mix issues and other activities taking place during the medication round (e.g. ward rounds).

High perceived workload, inadequate skill mix and particularly low staffing were major themes interconnecting the majority of errors. Low staffing was reported to be common place and appeared chronic in some cases, with causes including sickness, annual leave and wards acting as a staff “donor” (N11, > 5 years) to others requiring personnel. As a result, some staff reported working longer hours or being left on their own as the only qualified nurse during shifts, adversely affecting workload and nurse mental state.

Many participants felt that using agency nurses in response to staff shortages added to their workload and mental burden due to their perceived deficiencies in skill level and lack of familiarity with the ward, its patients and practices. One nurse felt she could not use agency staff for support when faced with a knowledge deficit:

“… which is another issue, agency staff only. They don’t know the ward, they don’t know the patients, so you’re the only qualified with three or four agency staff only, who don’t have the ward, who don’t know the patients. So you can’t rely on them. All you need to do is to rely on yourself, you know?”

(N08, 1–5 years)

#### The medicines

Medicines that sounded and/or looked a-like were discussed as contributory factors to error on four occasions, with three involving mix-ups between semi-sodium and sodium valproate. Four participants also described how storage of multiple pre-prepared depot syringes and other medicines (mainly valproate) next to each other contributed to selection errors.

Nursing staff emphasised how easy it was for them to mistake valproate products for one another, with one more experienced nurse describing how similarities in how they are written contributed to a skill-based slip:

“… because, you know, there’s only the ‘semi’ different and it was the same amount, same dose, you know, the same amount, same times and everything, it [sodium valproate] did look very much, you know, very similar [to semi sodium valproate] …”

(N03, > 5 years)

#### The medicines administration task

Challenging ward conditions appeared to affect the perceptions of nursing staff towards the medicines administration task. Participants described rushing medicines administration in order to carry out other tasks or to prioritise a competing demand (e.g. patient needs), with one experienced nurse reporting doing so because they felt isolated in the clinic room and out of control from the rest of the ward, resulting in medications being given to the wrong patient.

“… the reason that I was hurrying was because I wanted to get out of the clinic. I just wanted to make sure the medication was done and dusted, and then you can get back onto the ward. Because when you’re in the clinic, […] you don’t really know what’s going on, on the ward. And on a ward like [Ward name], it’s…you need to be aware of what’s going on at all times.”

(N11, > 5 years)

There was evidence of deficiencies relating to medicine double checking practices as well as patient identification. Although double checking was in place as a defensive barrier to error, MAEs occurred during both single (majority of medicines) and dual practitioner administration (e.g. controlled drugs, student nurses, depots, use of ‘runners’ to administer medication). For dual practitioner errors, examples included cases where either double checking did not take place, both staff members involved made the same mistake or information was miscommunicated between parties. Despite this, one nurse mentioned the protective effects of cross-checking for patient safety:

“Yeah there is [cross checking each other’s work] at times because like say sometimes things [medications] get changed from ward round but only one nurse is in there and then it might not always get handed over, but then it can be a good time for them to hand over that medication might have changed or things like that. It sort of jogs your memory sometimes.”

(N18, < 1 year)

#### Latent (wider organisational) failures

Whilst not identified as frequently as error/violation provoking conditions by participants, latent failures were reported as underpinning the emergence of staffing, skill mix and workload issues. One nurse suggested that the ward shift roster system was partly responsible for low staffing on their unit, and more than one participant commented that it was difficult for managers to identify staff replacements and for agencies to fill staff vacancies leading to skill mix and staffing problems. There were also suggestions that nurses were not allocated ‘protected time’ to administer medications on the ward and that scheduling ward rounds and patient meetings at the same time as medicines administration depleted available staff and increased workload.

## Discussion

To our knowledge, this is the first study to explore the causes of specific MAEs in a mental health hospital using in-depth qualitative interviews. Each MAE was reported to be caused by a combination of local error/violation provoking conditions relating to the patient, the health care team, the working environment, the medicines administration task, the individual nurse and the particular medicines involved, with wider organisational latent failures underpinning the emergence of many of these local conditions.

### Implications of findings

This study has brought into sharp focus the complexity of medicines administration on mental health wards and builds significantly on earlier work in the context of patient safety [15, 16, 21–23, 28, 29]. It is apparent that medicines administration regularly takes place in physically and cognitively demanding circumstances that could compromise defensive barriers and promote the occurrence of MAEs, and we have identified factors influencing the safety of medicines administration processes which are unique to this environment. We gathered in-depth accounts from nurses with different levels of experience who reported making errors across different mental health specialities. Our findings suggest that newly qualified staff might be particularly vulnerable and require additional support as a defensive barrier to help them practise safely and with confidence. The knowledge of the underlying causes of MAEs generated by this study will be an important contributor in the ongoing agenda to enhance mental health service quality [41, 42], and in informing the optimisation and evaluation of ward based medicines management services.

Whilst there are some similarities between the findings of our study and in-depth research of MAE causation in general hospitals across areas such as low staffing, interruptions, high workload and task management/double checking deficiencies [27, 30], our study has identified more context specific targets such as the impact of lone working and patient-related needs or behaviours (e.g. demands, aggression, lack of engagement).

A number of themes emerged as influential antecedents to multiple errors discussed in this study, and were also found to be linked to other causative factors. These are described below. However, it is important to recognise that whilst each theme might individually be isolated and mitigated to reduce the risk of error, future efforts to develop remedial interventions could consider a multifaceted approach to maximise positive impact as the MAEs in this study were often caused by interacting factors across multiple themes [27, 30, 43].

### Low staffing and inadequate skill mix

These themes preceded most MAEs and have emerged as quality measures in mental health with guidance being produced in the UK [44, 45]. There are a number of tools available to assess staffing levels [46] but there remains a need for those tailored to the mental health setting [47, 48]. Although the use of temporary staff in the mental health setting is a long-standing issue [49], with agency staff associated with a high cost burden [45], our study adds an important perspective to this narrative if nurses consider agency staff as creating problems in the medicines safety context. Mental health organisations are now considering more creative options to manage qualified nursing shortages [50]. However, the evidence of impact of staffing in relation to medicines safety is limited [48, 51–52] and further research could therefore measure, understand and mitigate these influential causative factors.

### Disruptive and distracting working environments

The busy working environment, and in particular distractions and interruptions, were highly influential in skill-based slips and lapses. These factors feature prominently in earlier studies of error causation in mental health [22, 23, 28, 29] but our findings add much needed contextual detail, and place more emphasis on patient-led causes of distractions and interruptions compared to evidence from general hospitals [27, 30] which could be crucial for the design of remedial interventions [53].

### Individual stress/pressure and task management

This study has emphasised the mental demands of the nursing role and the important influence this has on how nurses prioritise and carry out their duties. Previous studies have highlighted significant workplace stress for mental health nurses [54] and how mental workload can impact prescribing [33] and medicines administration safety in general hospitals [30, 55]. This indicates a need for more in-depth understanding of how factors such as patient advocacy, risk/benefit judgement and other personal and environmental circumstances affect decision-making in the medicines administration context in mental health. It may be useful to utilise techniques such as observation and hierarchical task analysis to characterise and predict the risks to medicines administration safety for nurses on the mental health ward, as seen in general hospitals [56, 57].

### Communication

We identified communication deficiencies that were both common to other settings (e.g. illegible prescriptions) but also unique to mental health settings (e.g. between nurses and ‘runners’ [22, 23, 16]). Communication deficiencies appear as frequent contributors to error in incident report studies in mental health hospitals [22, 23, 28, 29] though detail as to their nature which we identified is rarely provided. Whilst electronic prescribing and medicines administration systems are being introduced to improve medicines safety, there is evidence that nursing documentation discrepancies increase [58], and to our knowledge there have been no published evaluations of the impact of such systems in the mental health setting to date. Understanding and improving verbal communication between colleagues and with patients during medicines administration in the context of drug safety appears to be a subject of limited research [43, 59].

### Strengths and limitations

Strengths of this study include a focus on obtaining relevant data by restricting participant accounts to only examples in which they were directly involved. We used established data collection and analysis methods, though we did not triangulate with other methods such as observation which may help distinguish between what people say and what actually occurred. The research team also noted emerging data saturation at approximately the half way point of interviews which may have helped to ensure adequate coverage of the important contributory factors to MAEs.

The possibility of recall and hindsight bias cannot be excluded particularly as we asked participants identified from incident reports to recount events from up to 6 months previously, although the risk was minimised by asking participants to discuss events occurring within recent memory or those they could remember clearly. The risk of social desirability bias [60] may have been minimised as CIT was used to focus on actual actions and behaviours of participants, and attribution bias [61] may have also been minimised as the experience of the data collectors was that participants accepted their measure of responsibility for MAEs or near misses, and did not readily blame others.

## Conclusion

This study found that MAEs occurring in a UK mental health NHS hospital are each caused by a combination of interconnecting local error provoking conditions and latent ‘systems’ failures. Key antecedents to multiple types of MAE and thus central contributors to error were identified and included low staffing, inadequate skill mix, challenging ward environments (including patient factors), interruptions and distractions and communication problems. Future research efforts should be directed toward developing, testing and evaluating practical interventions designed to target these known causative factors, in order to offer informed recommendations to improve front line clinical services for the benefit of patients through reduced MAE rates.

## Supporting information

S1 Appendix (DOCX)
