# Supplementary material for: What causes medication administration errors in a mental health hospital? A qualitative study with nursing staff
Source: PLoS One. 2018 Oct 26;13(10):e0206233. doi: 10.1371/journal.pone.0206233 (PMC6203370; doi:10.1371/journal.pone.0206233)
Supplement: S1 Appendix — (DOCX) [file pone.0206233.s001.docx]

**Supplementary File – Appendix (S1 Appendix)**

**INTERVIEW SCHEDULE**

**What causes medication administration errors in a mental health hospital? A qualitative study with nursing staff**

(Please note: this interview schedule has been modified from the original for the purposes of publication)

Little is known about the causes of medication errors in mental health hospitals. The purpose of this interview is to discuss the occasions where an error was noticed either during or after you administered medication for inpatient(s) in [name] hospital, and to find out why you think these errors occurred. The area of interest is what events or circumstances led up to the error or near miss, rather than how it was handled afterwards.

Your right to confidentiality is protected at all times. We will not allow you to be recognised from any information we analyse or report from this interview. Details of patients or colleagues involved in any events that you describe are not required; if these are mentioned their details will be immediately removed from all records.

The interview will last between 30minutes to 1 hour, and will be audio-recorded unless you decide that you would not like to be audio-recorded. You can request that the recorder be turned off at any point during this interview. The [data] will be kept in a secure location for ten years after the study is complete and then destroyed.

I will begin the interview by getting some details about you and your background, followed by a discussion about the administration errors I asked you to think about. I will then finish with some more general questions around this topic.

Do you have any questions before beginning the interview?

**Part One - background**

Can you tell me a little about your professional background?

- Name and gender
- Training background (including relevant medication related training)
- Years qualified
- Current job title
- Speciality

Are you already involved in another research study?

**Part Two – the administration errors**

In the letter I sent you, I asked if you could consider one or more errors or near misses that occurred whilst you were administering medication. Could you please tell me about these errors?

- **Areas to be covered**
  - Nature of error
    - Type of error (dose, timing/frequency, interaction, contraindication, route, omission, preparation, dosage form, un-authorised drug, administration technique)
    - Medication involved
    - What was being treated and its severity
    - Did the error reach the patient?
      - Consequences for the patient
    - How was the error discovered
  - Circumstances of error
    - Time of day, day of week
    - Ward where error occurred, length of time on ward
    - Physical and mental state at time of error/near miss
    - Anyone else involved – their role and impact
    - General workload
    - Supervision – trust, was help sought?
    - Stage of patient journey through hospital
    - Patient particulars (without names) – your patient? Seen patient before? Relationship with patient? Patient complexity?

**Interview top tips**

- Difficulty with recall? – ask to consider error they made that has really made a deep impression on them and their practice
- Use prompts to gather more detailed information about the error
  - Could you tell me more about that?
  - Could you give me a more detailed description of what happened?
  - Tell me what you are thinking about
  - You mentioned ……. – what do you mean by that?
  - You mentioned ……. before, could you talk a more about that?
  - Why did you hesitate just then?

Many thanks for taking the time to help us with this research study. Your contribution has been extremely valuable. If you wish we can send you a copy of your interview transcript. Please contact us if you would like to receive a summary of the findings of this research study. In the meantime please feel free to contact us using your information leaflet if you have any questions or other issues to discuss.

**Part Three – conclusion**

Is there anything that you would like to talk about?

Is there anything that you would like to go back and talk about?

Switch off tape recorder

**Part Two – the administration errors**

- **Areas to be covered**
  - Reasons for making the error
    - Slip, lapse, mistake or violation
    - Communication difficulties, team working, work environment, task support, knowledge, training and experience, support, procedures/medication charts
    - Knowledge / opinion toward task related procedures and medications
  - Attitude toward the error
    - Has this happened before? Did you do things differently this time and why?
    - How did it make you feel?
      - Why and for how long?
      - Did you alter your practice as a result?
    - Could anything have prevented the error / near miss?
      - Refer back to reasons above to ensure coverage
